# The Comparison in the Microstructure and Mechanical Properties between AZ91 Alloy and Nano-SiCp/AZ91 Composite Processed by Multi-Pass Forging Under Varying Passes and Temperatures

**DOI:** 10.3390/ma12040625

**Published:** 2019-02-20

**Authors:** K.B. Nie, J.G. Han, K.K. Deng, X.J. Wang, C. Xu, K. Wu

**Affiliations:** 1 College of Materials Science and Engineering, Taiyuan University of Technology, Taiyuan 030024, China; hanjgang@hotmail.com (J.G.H.); dengkunkun@tyut.edu.cn (K.K.D.); 2 Shanxi key laboratory of advanced magnesium-based materials, Taiyuan University of Technology, Taiyuan 030024, China; 3 School of Materials Science and Engineering, Harbin Institute of Technology, Harbin 150001, China; xjwang@hit.edu.cn (X.J.W.); xuchao@vos.nagaokaut.ac.jp (C.X.); wukunhit@163.com (K.W.); 4 Research Center for Advanced Magnesium Technology, Nagaoka University of Technology, Nagaoka 940-2188, Japan

**Keywords:** AZ91 alloy, SiC nanoparticles, Multi-pass forging, Microstructure, Mechanical properties

## Abstract

In this study, both AZ91 alloy and nano-SiCp/AZ91 composite were subjected to multi-pass forging under varying passes and temperatures. The microstructure and mechanical properties of the alloy were compared with its composite. After six passes of multi-pass forging at a constant temperature of 400 ℃, complete recrystallization occurred in both the AZ91 alloy and composite. The decrease of temperature and the increase of passes for the multi-pass forging led to further refinement of dynamic recrystallized grains and dynamic precipitation of second phases. The grain size of the nano-SiCp/AZ91 composite was smaller than that of the AZ91 alloy under the same multi-pass forging condition, which indicated that the addition of SiC nanoparticles were beneficial to grain refinement by pinning the grain boundaries. The texture intensity for the 12 passes of multi-pass forging with varying temperatures was increased compared with that after nine passes. The ultimate tensile strength is slightly decreased while the yield strength was increased unobviously for the AZ91 alloy with the decrease of temperature and the increase of the passes for the multi-pass forging. Under the same condition of multi-pass forging, the yield strength of the composite was higher than that of the AZ91 alloy due to the Orowan strengthening effect and grain refinement strengthening resulting from externally applied SiC nanoparticles and internally precipitated second phases. By comparing the microstructure and mechanical properties between the AZ91 alloy and nano-SiCp/AZ91 composite, the strength-toughness properties of the composites at room temperature were affected by the matrix grain size, texture evolution, SiC nanoparticles distribution and the precipitated second phases.

## 1. Introduction

As the lightest commercially available structural metal, magnesium possesses low density and high specific strengths over other metallic metals [1]. The corresponding applications of magnesium alloys in the automotive, transportation and electronic industries have risen significantly owing to the increased demand of fuel economy, light-weighting, and performance [2,3]. However, compared with the aluminum alloys and steels, magnesium alloys are uncompetitive due to their inferior high-temperature mechanical properties, and corrosion and wear resistances [4]. Besides, the hexagonal close packing (hcp) structure of magnesium alloys strongly affects the plastic deformation, resulting in relatively low strength and poor room temperature ductility [5]. In order to improve the mechanical properties of magnesium and its alloys, one of the effective methods is to develop magnesium matrix composites [6,7,8]. When one or more reinforcements are introduced into the magnesium matrix, it usually helps to improve the mechanical properties such as high strength, superior creep and wear resistance at elevated temperature. Therefore, more and more attention has been paid on the magnesium matrix composites with low density and superior specific mechanical properties [9,10,11,12]. Since the addition of micron-sized ceramic particles deteriorates the ductility of matrix alloy, many attempts have been made to develop magnesium matrix nanocomposites by substituting micron-sized particles by nano-sized particles [13,14,15,16]. Extensive researches have shown that the introduction of inexpensive low volume fraction of nanoparticles into magnesium matrix assists in achieving simultaneous enhancement in strength and ductility without adversely affecting the density of the material. In our previous study, cost-effective fabrication technology involving in semisolid stirring and ultrasonic infiltration has been developed to incorporate and disperse nano-sized SiC particles homogeneously in magnesium alloy [17]. 

With regard to numerous studies regarding the fabrication of magnesium matrix nanocomposites, limited attention has been paid on the secondary thermo mechanical processing of as-cast nanocomposites [18,19,20]. For example, Liu et al. applied hot rolling to the nano-SiCp/AZ31 composite, resulting in improved yield strength [18]. Choi et al. investigated the effect of hot extrusion on the nano-SiCp/Mg composites and reported that ultimate tensile strength, yield strength and ductility were improved as compared to the matrix alloy [19]. Our previous study investigated the effect of hot extrusion on the nano-SiCp/AZ91 composite and reported the occurrence of extensive dynamic recrystallization (DRX) and significant refinement of matrix microstructure [20]. Therefore, the quality of magnesium matrix nanocomposite can be further improved by the application of thermal deformation. Meanwhile, recent studies have shown that compared with conventional thermal deformation, it is more effective to produce bulk fine-grained magnesium alloys using severe plastic deformation (SPD) [21,22,23,24]. In general, the grain size can be reduced to 1 μm when the SPD techniques are applied at room temperature. However, with respect to magnesium alloy the SPD processing is usually performed at elevated temperatures due to its low ductility of hexagonal structure [25]. Among various SPD techniques, multi-pass forging is frequently used since this procedure is capable of producing homogeneous fine-grained microstructures in bulk materials with large dimensions [26,27]. Furthermore, the thickness and diameter of the sample can be kept after multi-pass forging, which is different from conventional thermal deformation. Currently, the multi-pass forging has been successfully applied to obtain sub-micrometer or nanometer grains in pure metals and metallic alloys [28,29]. Xia et al. reported that after multi-pass forging the microstructure with the grain size of 1.3 μm was homogeneous and ultimate tensile strength was improved [29]. The multi-pass forging led to the decrease in the grain size of Mg-Gd-Y-Nd-Zr alloy and significant improvement in both the yield and ultimate strength. However, the addition of SiC nanoparticles to the AZ91 matrix could yield significant differences in the as-processed microstructures even for the same equivalent strains [30]. Therefore, a comparison of the microstructures between AZ91 alloy and nano-SiCp/AZ91 composite processed by multi-pass forging under varying passes and temperatures has a great significance. Although, some studies have already investigated the multi-pass forging of conventional micro-particles reinforced magnesium matrix composites and magnesium alloys [31,32], a systematic comparison between the microstructures between AZ91 alloy and nano-SiCp/AZ91 composite processed by multi-pass forging under varying passes and temperatures has not been given yet. In addition, a comparative study on the development of dynamic precipitated second phases for the AZ91 alloy and nano-SiCp/AZ91 composite is missing from the literature. The present work fills this gap and provides the evolution of the grain size and the precipitation of second phases of the AZ91 alloy and nano-SiCp/AZ91 composite fabricated by multi-pass forging under varying passes and temperatures. In addition, the change of the microstructure as a function of the number of passes was also monitored and the differences observed for the AZ91 alloy and nano-SiCp/AZ91 composite are discussed. The mechanical properties of the samples were measured and correlated to the microstructure. 

## 2. Material and Methods

### 2.1. Sample Processing

Magnesium alloy AZ91 with a chemical composition (wt %) of Mg-9.07Al-0.68Zn-0.21Mn (Northeast Light Alloy Company Limited, Harbin, China) was chosen as matrix alloy. Nano-sized SiC particles with an average dimension of 60 nm (Hefei Kaier Nanometer Energy & Technology Company Limited, Hefei, China) were employed as raw materials, which possess adequate properties. The preparation steps for the magnesium matrix nanocomposites involved in semisolid stirring and ultrasonic infiltration. The used fabrication process has been described in detail in Ref. [17]. During the fabrication, AZ91 alloy melt was first kept at a temperature of 590 °C to make the melt in semi-solid state. Then, SiC nanoparticles with volume fraction of 1% were added using mechanical stirring under a shielding gas of CO_2_/SF_6_. Next, the melt containing SiC nanoparticles was heated to 700 °C and subjected to ultrasonic vibration. Finally, a mixture of magnesium and SiC nanoparticles was poured into a metal mold (preheated to 450 °C) and solidified under pressure. Note that there was no ultrasound applied during solidification.

### 2.2. Processing of the Samples by Multi-Pass Forging

Before multi-pass forging solution treatment at 415 °C for 24 h was carried out on both the as-cast AZ91 alloy and nanocomposite to minimize the influence of Mg_17_Al_12_ phase. Rectangular billet specimens with a size of 30 × 30 × 60 mm were prepared by an electrical discharge machine. The temperatures for the multi-pass forging ranged from 250 °C to 400 °C at a constant punch velocity of 15 mm s^−1^. The sample size remained unchanged on the whole after multi-pass forging although the loading direction was rotated to 90° between passes. All the specimens were heated to the desired temperatures for multi-pass forging using a resistance furnace with the aim of achieving a uniform temperature distribution. The strain for each pass was 0.693 during the multi-pass forging. Graphite-based mixture was selected as lubricant for the multi-pass forging under varying passes and temperatures.

### 2.3. Study of the Phase Composition and the Microstructure

To disclose the microstructural characteristics, microstructures of the AZ91 alloy and nanocomposites were examined by means of optical microscopy (OM, Shanghai Optical Instrument Factory, Shanghai, China), scanning electron microscopy (SEM, Tescan, Brno, Czech Republic), and transmission electron microscopy (TEM, JEOL Ltd., Tokyo, Japan). Following the standard metallographic procedures, samples for metallographic observation and SEM were prepared by the polishing machine and then etched in acetic picral [5 mL acetic acid + 6 g picric acid + 10 mL H_2_O + 100 mL ethanol (95%)]. Samples for electron backscattered diffraction (EBSD) were prepared by electrical polishing in solution consisting of ethanol and phosphoric acid. The grain size was analyzed by Image-Pro Plus software. In order to detect the distribution of SiC nanoparticles and the precipitated phases, samples for TEM were manual grinding, sliced and ion beam thinned following the sample preparation procedures. The texture change of nano-SiCp/AZ91 composites is also tested by EBSD.

### 2.4. Tensile Test

In uniaxial tension experiment, mechanical properties of both the AZ91 alloy and the nanocomposites reinforced by SiC nanoparticles were determined using an Instron-1186 universal testing system. The obtained flat dog-bone samples had a gage size of 15 × 6 × 2 mm, which were machined from the sample perpendicular to the last forging axis. The tensile tests on the samples were conducted at an initial strain rate of 8.33 × 10^−4^ (s^−1^) at room temperature. The strength values and elongation reported in the present work were obtained based on three repeated tensile tests.

## 3. Results and Discussion

### 3.1. Microstructure after Multi-Pass Forging with Decreasing Temperatures

**Figure 1:**
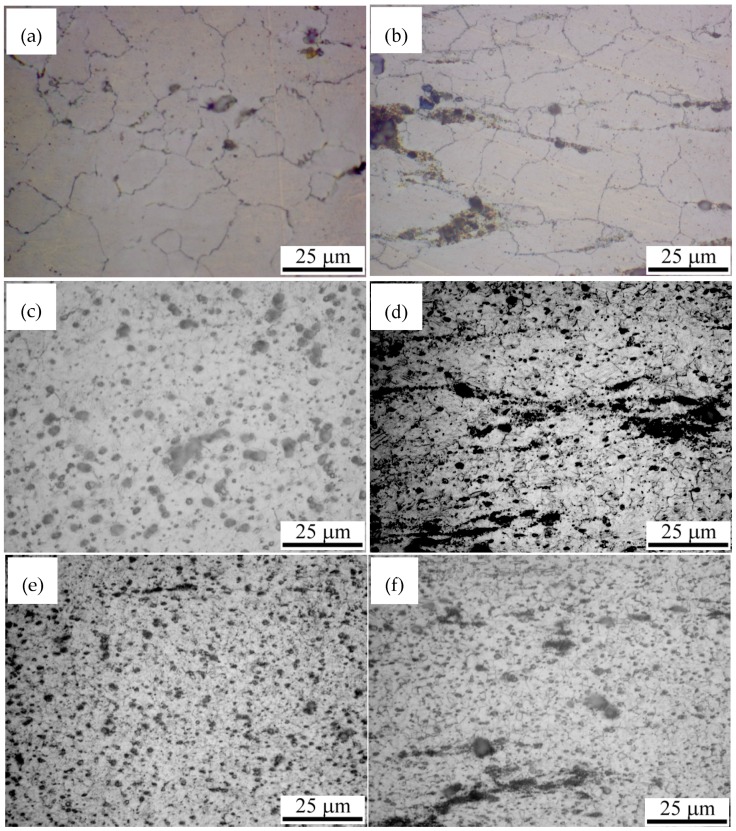
OM images of AZ91 alloy after multi-pass forging for (**a**) six passes, (**c**) nine passes, (**e**) 12 passes, and nano-SiCp/AZ91 composites after multi-pass forging for (**b**) six passes, (**d**) nine passes, (**f**) 12 passes (six passes–400-6P, nine passes–400-6P + 350-3P, 12 passes–400-6P + 350-3P + 300-3P).

**Figure 2:**
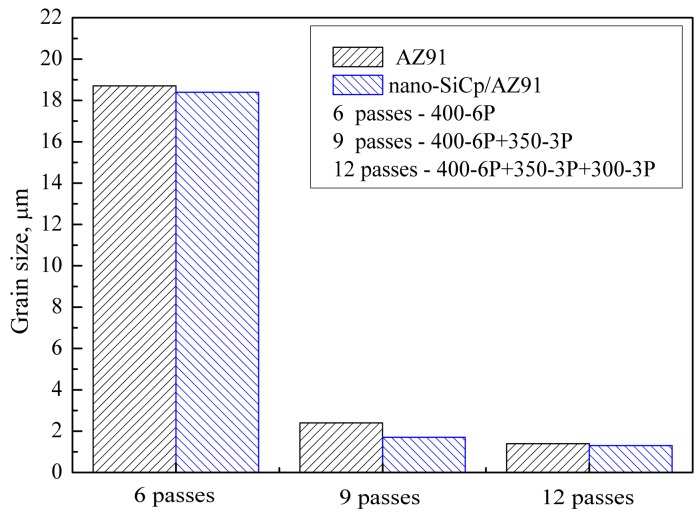
Grain sizes of AZ91 alloy and nano-SiCp/AZ91 composites after multi-pass forging for different passes.

The OM images for the AZ91 alloy and nano-SiCp/AZ91 composite processed by different passes of multi-pass forging with decreasing the temperatures are shown in Figure 1. Figure 2 gives the grain sizes of AZ91 alloy and nano-SiCp/AZ91 composites after different passes of multi-pass forging. It can be observed from Figure 1a,b that both the grains for the AZ91 alloy and composite after six passes of multi-pass forging at a constant temperature of 400 °C are significantly finer than that of the as-cast counterparts [17], which indicates the occurrence of complete recrystallization. Upon decreasing the temperature from 400 °C to 350 °C during multi-pass forging, as shown in Figure 1c,d, both the grains for the AZ91 alloy and composite after six passes at 400 °C and three passes at 350 °C are further refined. Besides, the amount of the precipitates after nine passes of multi-pass forging is more than that after six passes. As given in Figure 2, grain sizes for the AZ91 alloys processed by six and nine passes of multi-pass forging are 18.7 and 2.4 μm, respectively. In contrast, the grain sizes for the composites processed by six and nine passes of multi-pass forging are 18.5 and 1.7 μm, respectively. When the temperature for multi-pass forging decreases from 400 °C to 300 °C, as given in Figure 1e,f, there is no obvious grain refinement for the AZ91 alloy and composite after six passes at 400 °C, three passes at 350 °C and three passes at 300 °C. The grain sizes for the AZ91 alloy and composite after 12 passes of multi-pass forging are 1.4 and 1.3 μm, respectively. The subsequent three passes of multi-pass forging at 300 °C is effective for the precipitation of second phases compared with that after nine passes as can be seen in Figure 1c,d. With the increase of passes for multi-pass forging under varying temperatures, dynamic recrystallization continues to occur due to the increase of cumulative deformation, resulting in further grain refinement. On the other hand, during the multi-pass forging the increase of precipitated second phases near the grain boundaries as the temperature decreases and the addition of SiC nanoparticles can pin the recrystallized grain boundaries and prevent recrystallized grain growth. In the same process condition, the average grain size of the nano-SiCp/AZ91 composite matrix is smaller than the AZ91 alloy, which further confirms that the addition of SiC nanoparticles is beneficial to hinder the growth of recrystallized grains. Compared with the AZ91 alloy after three passes at 400 °C, three passes at 350 °C and three passes at 300 °C [33], the grain size for the present AZ91 alloy after six passes at 400 °C and three passes at 350 °C is slightly increased. This is due to the fact that the wider range of temperature reduction can lead to finer second phase precipitated near the grain boundaries, which is beneficial to hinder the growth of recrystallized grains. When the temperature range for the multi-pass forging is the same, it can be found that with the increase of passes the average grain size of the alloy after six passes at 400 °C, three passes at 350 °C and three passes at 300 °C is reduced and the uniformity of the structure is improved as compared to the alloy after three passes at 400 °C, three passes at 350 °C and three passes at 300 °C. With regard to the nano-SiCp/AZ91 composite, it can be also found that the grain size after six passes at 400 °C and three passes at 350 °C is decreased compared with the composite after three passes at 400 °C, three passes at 350 °C and three passes at 300 °C [34]. This is mainly due to the fact that the higher temperature of multi-pass forging is beneficial to further break up the aggregation of nanoparticles, leading to the increase in the number of dispersed SiC nanoparticles which can hinder the growth of the recrystallized grains. As the temperature ranges from 400 °C to 300 °C, with increasing the passes of the multi-pass forging, the average grain size of the composite after 12 passes is further decreased and the homogeneity of microstructure is improved than that after nine passes.

**Figure 3:**
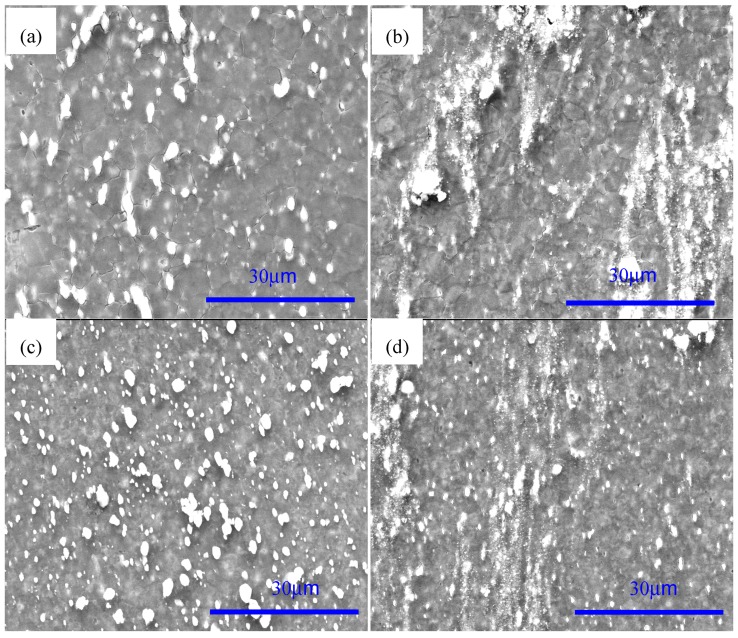
SEM images of AZ91 alloy after multi-pass forging for (**a**) nine passes and (**c**) 12 passes, and nano-SiCp/AZ91 composites for (**b**) nine passes and (**d**) 12 passes.

**Figure 4:**
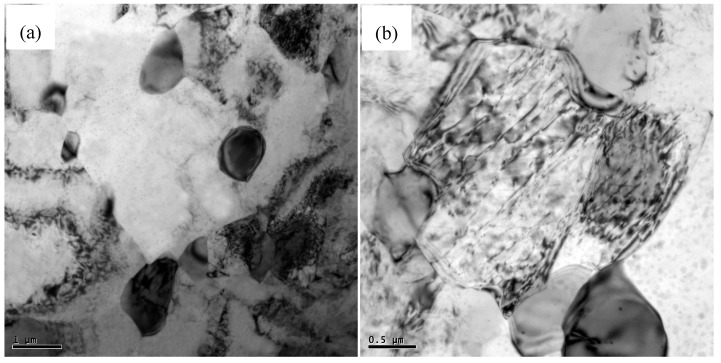
TEM micrographs at high magnification of AZ91 alloy after multi-pass forging for 12 passes (400-6P + 350-3P + 300-3P): (**a**) DRX grains and coarse precipitated phase, (**b**) DRX grains and dislocation.

**Figure 5:**
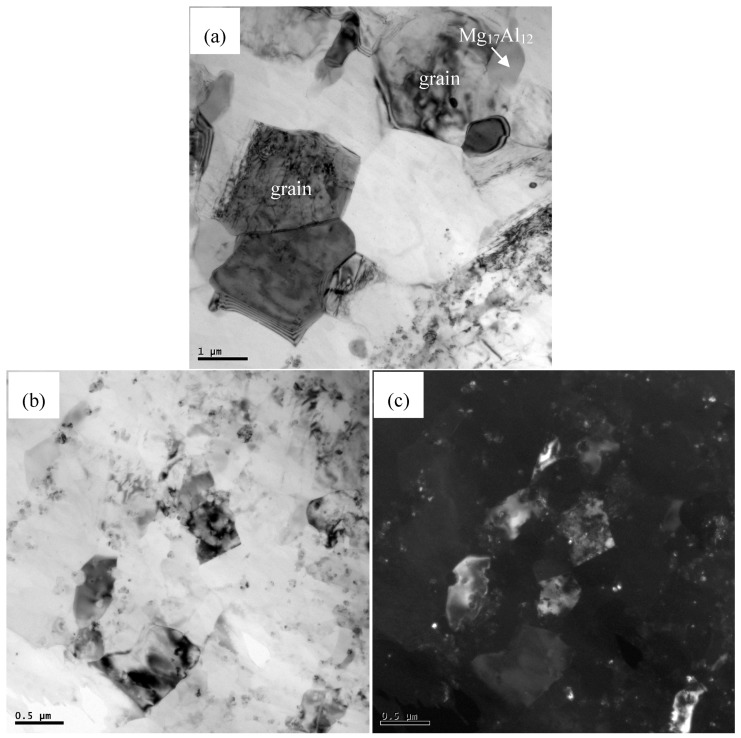
TEM micrographs of nano-SiCp/AZ91 composite after multi-pass forging for 12 passes (400-6P + 350-3P + 300-3P): (**a**) DRX grains and precipitated phase, (**b**,**c**) distribution of SiC nanoparticles.

Figure 3 shows the SEM images of AZ91 alloy and nano-SiCp/AZ91 composites after multi-pass forging for nine passes and 12 passes. For the AZ91 alloy processed by nine and 12 passes of multi-pass forging, as shown in Figure 3a,c, the grain size is refined with the increase of the passes and the decrease of the temperature. The amount for the precipitated second phases is significantly increased and the size of some precipitates exhibits substantial increase. It can be seen from Figure 3b,d that there is also an obvious increase in the amount of the precipitated phases for the nano-SiCp/AZ91 composite after 12 passes of multi-pass forging. Figure 4 shows further observation of AZ91 alloy after by TEM after multi-pass forging for 12 passes. It can be observed from Figure 4a that the recrystallized grains of AZ91 alloy after 12 passes of multi-pass forging at varying temperatures are significantly refined as compared to that after six passes of multi-pass forging at a constant temperature of 400 °C. The precipitated phases can be found along the grain boundaries of the recrystallized grains, which is consistent with the OM observations (Figure 1e). At high magnification, as shown in Figure 4b, the second phase is located along a single recrystallized grain that would hinder the growth of the dynamic recrystallized grain. Figure 5 gives the TEM observation of nano-SiCp/AZ91 composites after multi-pass forging for 12 passes. It can be seen from Figure 5a that there are some recrystallized grains and the second phase Mg_17_Al_12_ exists at the grain boundaries within the free-nanoparticles zone. Through the bright field TEM observation as shown in Figure 5b, the overall distribution of SiC nanoparticles is relatively uniform and the dense- nanoparticles zone possess low dislocation density. Compared with the free-nanoparticles zone (Figure 5a), the dark field TEM image (Figure 5c) reveals that the recrystallized grain size of the dense-nanoparticles zone is significantly reduced. This further indicates that the addition of SiC nanoparticles facilitates the grain refinement of the nanocomposite matrix.

**Figure 6:**
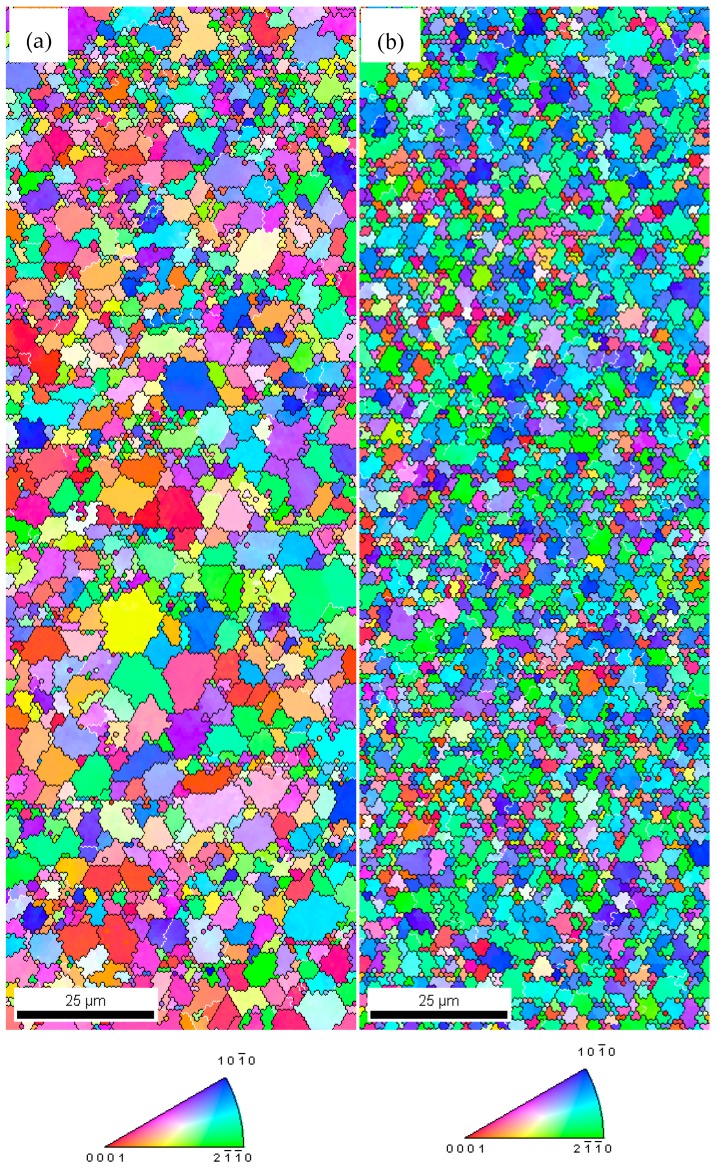
EBSD analysis of nano-SiCp/AZ91 composite after multi-pass forging for (**a**) nine passes (400-6P + 350-3P) and (**b**) 12 passes (400-6P + 350-3P + 300-3P).

**Figure 7:**
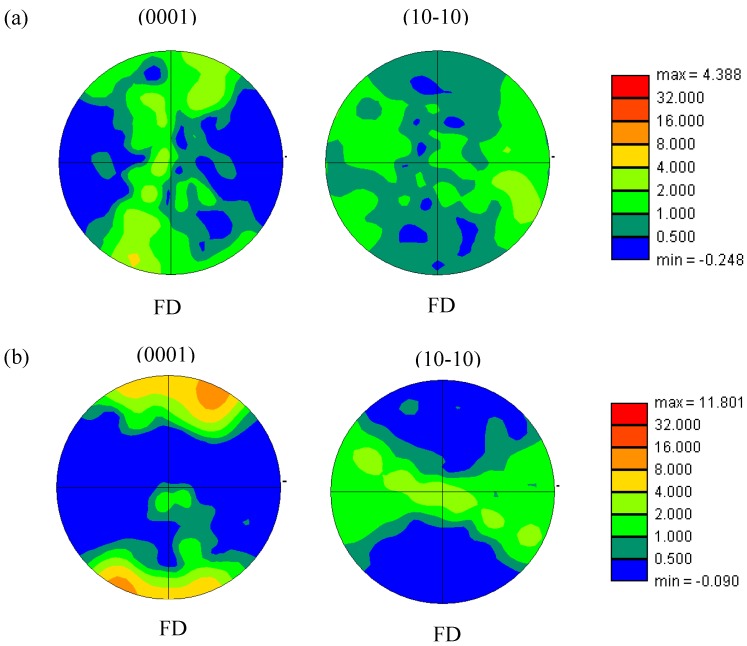
Pole figures of nano-SiCp/AZ91 composite after multi-pass forging: (**a**) for six passes (400-6P), (**b**) for 12 passes (400-6P + 350-3P).

The EBSD analysis of the nano-SiCp/AZ91 composite after nine and 12 passes of multi-pass forging is shown in Figure 6. It can be seen from Figure 6 that the grain size of the nano-SiCp/AZ91 composite after 12 passes is smaller than that after nine passes, which is consistent with the previous OM observation. However, the overall grain size of the nanocomposite obtained by EBSD is larger than the average grain size observed by OM. This is due to the fact that the dense-nanoparticles zone would be considered as a large grain in the EBSD analysis. Besides, as shown in Figure 6a, all the grains in the composite after nine passes of multi-pass forging are high-angle grain boundaries, which indicate that the recrystallization is relatively complete. At the same time, there are also some finer recrystallized grains within the dense-nanoparticles zone, which is consistent with the previous SEM observation. Similar phenomenon can be observed for the composite with remarkably refined grain size after 12 passes of multi-pass forging. Figure 7 shows the texture tested by EBSD of nano-SiCp/AZ91 composite after multi-pass forging for nine passes and 12 passes. With the decrease of the pass and temperature for the multi-pass forging, the texture intensity for the 12 passes(400-6P + 350-3P + 300-3P) is significantly increased. 

### 3.2. Tensile Properties after Multi-Pass Forging with Decreasing Temperatures

**Figure 8:**
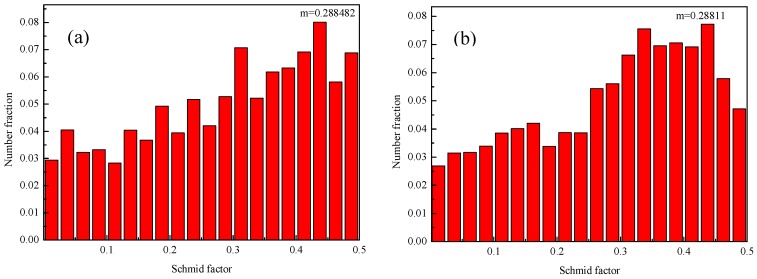
Schmid factors of nano-SiCp/AZ91 composites after multi-pass forging: (**a**) for 9 passes (400-6P + 350-3P), (**b**) for 12 passes (400-6P + 350-3P + 300-3P).

**Figure 9:**
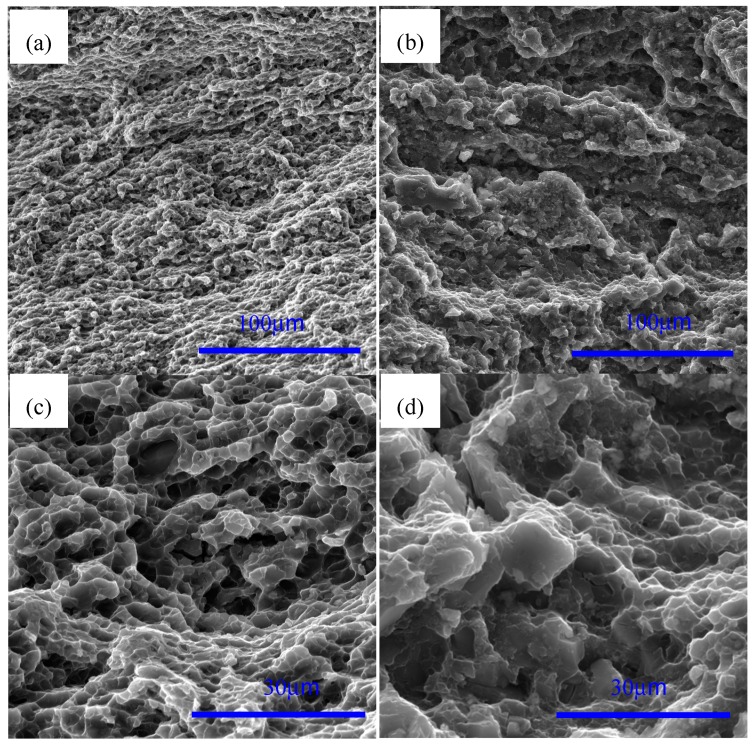
SEM images of fracture surface after multi-pass forging for 12 passes: (**a**,**c**) AZ91 alloy and (**b**,**d**) nano-SiCp/AZ91 composites.

Table 1 shows the mechanical properties of AZ91 alloy and nano-SiCp/AZ91 composites after multi-pass forging under varying passes and temperatures. It can be found from Table 1 that the addition of SiC nanoparticles has a significant effect on the tensile behavior of the AZ91 matrix alloy. As compared to the as-cast AZ91 alloy [17], the ultimate tensile strength and yield strength of the AZ91 alloy after multi-pass forging for nine passes and 12 passes are simultaneously improved due to the significant refined grains. With the decrease of temperature and the increase of the passes for the multi-pass forging, the ultimate tensile strength is slightly decreased while the yield strength is increased unobviously. Based on the microstructure observation for the AZ91 alloy (Figure 1 and Figure 2), the grain size is gradually reduced for the AZ91 alloy after multi-pass forging for nine passes, resulting in the improvement in the yield strength. In contrast, there is no obvious refinement for the grain size of the AZ91 alloy after nine and 12 passes of multi-pass forging, the change in the strength can be related to the growth of the second phase and the texture change. With regard to the nano-SiCp/AZ91 composites, the ultimate tensile strength and yield strength after multi-pass forging for six, nine and 12 passes are also increased compared to the as-cast counterparts. Besides, the grain size of the nano-SiCp/AZ91 composites after nine passes is smaller than that after six passes at constant temperature, leading to the increase in the yield strength. Under the same conditon of multi-pass forging, the yield strength of the composite is higher than that of the AZ91 alloy due to the Orowan strengthening effect and grain refinement strengthening. As shown in Figure 8, the difference in the average values for schmid factors between the nano-SiCp/AZ91 composites after multi-pass forging for nine passes (400-6P + 350-3P) and for 12 passes (400-6P + 350-3P + 300-3P) are not significant. But the component of the schmid factor which possesses larger values increases for the composite after 12 passes, this means that the material soften occurs. Thus, the yield strength of the composite after 12 passes is slightly decreased than that after nine passes. The yield strength of the composite after six passes at 400 °C, three passes at 350 °C followed with/without three passes at 300 °C is improved compared with the composite after three passes at 400 °C, three passes at 350 °C followed with/without three passes at 300 °C [34]. This is because the increased passes for multi-pass forging at higher temperature can improve the nanoparticle distribution and promote the grain refinement and the Orowan strengthening. The elongation of the sample AZ91 for 12 passes is higher than other alloys, which can be mainly attributed to the significantly refined grain size as shown in Figure 2. According to the added tensile fracture as shown in Figure 9, the high amount of dimple for the sample AZ91 after 12 passes are consistent with the higher elongation. SEM images of fracture surface of AZ91 alloy and nano-SiCp/AZ91 composite after multi-pass forging for 12 passes are shown in Figure 9. In general, the fracture characterization of Mg alloys is related to cleavage fracture or quasi-cleavage fracture due to their restricted dislocation slip systems. After multi-pass forging for 12 passes as shown in Figure 9a,c, the AZ91 alloy exhibits a ductile failure, which could be attributed to the grain refinement. In contrast, it is obvious that the number of dimples for the AZ91 alloy is greater than that of the nano-SiCp/AZ91 composite. There is no significant change in the grain size between the AZ91 alloy and the composite, so the different characterization of the tensile fracture can be mainly attributed to the addition of nano-sized SiC particles. The SiC nanoparticle dense zones are thought to play a major role in the decrease of elongation. Besides, the average diameter of the added SiC nanoparticles is 60 nm in the present work, and the interface between single SiC nanoparticle and matrix alloy is difficult to be observed on the tensile fracture surface.

### 3.3. Affecting Factors for the Strength-Toughness Properties of the Nano-SiCp/AZ91 Composites

According to the research regarding on the microstructure and mechanical properties of the AZ91 alloy and nano-SiCp/AZ91 composite, the strength-toughness properties of the composites at room temperature are affected by the matrix grain size, texture evolution, SiC nanoparticles distribution and the precipitated second phases. Since all the above affecting factors are interactive, it is necessary to give a comprehensive discussion of these influences on the mechanical properties. 

#### 3.3.1. Effect of Grain Size on the Strength-Toughness Properties

**Figure 10:**
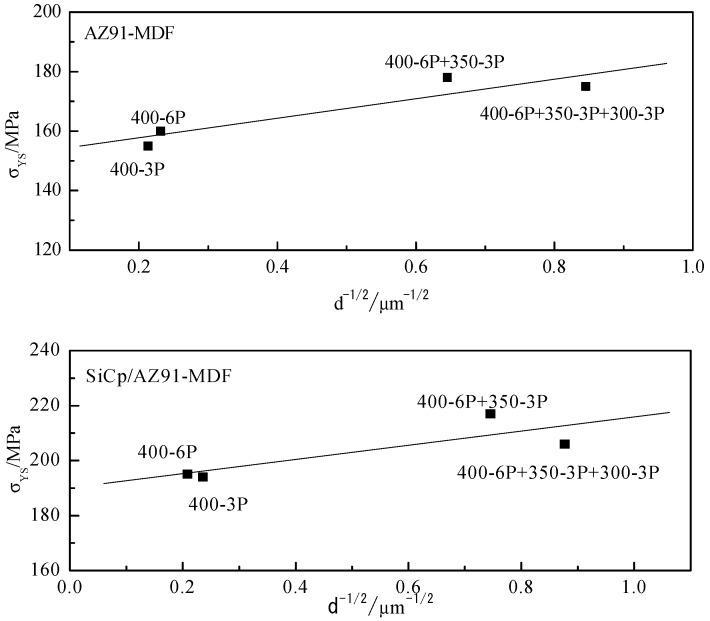
Relationship between yield strength σ_YS_ and inverse square root d^−1/2^ of grain size in AZ91 alloy and nano-SiCp/AZ91composites after multi-pass forging for different passes.

The finer grain size usually leads to higher plasticity and yield strength for the magnesium alloy. The increment in the plasticity due to grain refinement can be attributed to that of when the grain size is small with the same volume, the number of grains with favorable orientation would increase during the plastic deformation and the deformation can be more evenly distributed in the alloy. The grain boundary strengthening can be related to the grain boundaries which can block dislocation glide during deformation, resulting in dislocation tangle. The Hall-Petch relationship is used to describe the applied stress for further deformation [35]. In the current work, the relationship between yield strength σ_YS_ and inverse square root d^−1/2^ of grain size for AZ91 alloy and nano-SiCp/AZ91 composites after different passes are given in Figure 10. It can be found from Figure 10 that the yield strength of the AZ91 alloy and its composites after multi-pass forging for six passes at 400 °C meet the Hall-Petch formula to some extent. However, the yield strength value of the AZ91 alloy and its composites after nine and 12 passes of multi-pass forging deviates from the above Hall-Petch formula. In addition, the yield strength of the composite is higher than that of the AZ91 alloy with the same deformation condition. This is due to the initial grain sizes for both the AZ91 alloy and the composite being large, the yield strength would increase with decreasing the grain size. This indicates that the Hall-Petch strengthening is the main affecting factor for the nanocomposite. With regard to the alloy and the composites after nine and 12 passes of multi-pass forging under varying temperatures, the grain size is significantly reduced but the number of the precipitated phases around the grain boundaries is obviously increased. On the one hand, these precipitated phases can affect the recrystallized grains, and thus influence the final yield strength of the alloy and the composite. On the other hand, the dispersed nanoparticles can contribute to the Orowan strengthening. As for the nano-SiCp/AZ91 composite, the effect of the SiC nanoparticles distribution on the Hall-Petch strengthening and Orowan strengthening should be considered. In this case, the deviation from the above Hall-Petch formula exist for the alloy and the composites after nine and 12 passes of multi-pass forging under varying temperatures. Furthermore, complete recrystallization occurs for the composites after nine and 12 passes of multi-pass forging. The matrix in the composite is mainly comprised of high-angle grain boundaries and the orientation difference between adjacent grains is large. When one grain is in the direction favorable to dislocation slip, it is relatively difficult to active the dislocation sources for the adjacent grain. Since the Hall-Petch relationship is built on that the grain boundary prevents dislocation slip, the occurrence of grain boundary slip at room temperature deformation would relax the stress concentration caused by dislocation tangle [36]. This leads to the reduced stress for further slip of dislocation, thus affecting the strength of the composites.

#### 3.3.2. Effect of Texture on the Strength-Toughness Properties

Since magnesium has a hexagonal close-packed crystal structure, the slip system is limited and only the basal slip can be activated at room-temperature tensile deformation. So, the texture change after deformation will affect the mechanical properties of the AZ91 alloy and the composite. The effect of texture on the yield strength of a material can be expressed by the change in schmid factor m [37]:

(1) $$m=\tau_{c}/\sigma_{s}=\cos\lambda \cos\varphi$$

where $\tau_{c}$ is the critical shear stress, $\sigma_{s}$ is the tensile stress of the material, λ stands for the angle between the tensile stress axis and the slip direction, φ represents the normal angle between the tensile stress axis and the normal of slip plane. 

In our previous research and the present work, based on the influence of the addition of SiC nanoparticles on the texture evolution and the mechanical properties of AZ91 alloy and nano-SiCp/AZ91 composites, it shows that the addition of SiC nanoparticles does not change the texture type of matrix magnesium alloys but can change the texture intensity of the matrix. After one pass of multi-pass forging, there is strong basal texture in the alloy and the composite, which means that most of the basal plane is perpendicular to the initial forging direction. Besides, the direction of the tensile stress is always parallel to most of the basal planes for the tensile specimen when the tensile test is conducted perpendicular to the initial forging direction at room temperature. The values of the Schmid factor for the basal slip are small, which is not conducive to the basal slip system activation, contributing to the improvement in the yield strength of the composite after initial forging. With increasing the pass of the multi-pass forging at 400 °C, the basal plane of the composites is continuously deflected with the change in the direction of the applied load axial and the basal texture intensity of the composite decreases. This results in the basal deflection of more and more grains, the number of basal planes parallel to the tensile stress decreases during the room-temperature tensile test. However, with the increase of the pass and the decrease of temperature for the multi-pass forging, the texture intensity after 12 passes (400-6P + 350-3P + 300P) is increased compared with that after nine passes of multi-pass forging at 400 °C. The texture changes could also affect the angular relationship between the basal plane and the tensile direction.

However, there are still many discussions on the effects of texture on the strength of alloys and composites. It is generally believed that the yield strength of the alloy decreases as the Schmid factor increases. However, Kim et al. [38] showed that the texture softening can lead to the anti-Hall-Petch relationship for the magnesium alloy subjected to equal-channel angular pressing. For the current nano-SiCp/AZ91 composites, the average grain size after multi-pass forging for 12 passes (400-6P + 350-3P + 300-3P) is decreased while the yield strength decreases as compared to the composite after multi-pass forging for nine passes (400-6P + 350-3P). The difference in the average values for schmid factors between the composites after nine and 12 passes is not significant as given in Figure 8, but the component of the schmid factor which possesses larger values increases for the composite after 12 passes, resulting in the slight decrease of the yield strength. 

#### 3.3.3. Effect of Precipitated Second Phase on the Strength-Toughness Properties

The precipitation of the second phase during multi-pass forging may not only hinder the growth of dynamic recrystallized grains, but also affect the subsequent passes of multi-pass forging. Related studies have shown that when the size of the second phase is large, the grain refinement during severe plastic deformation will be accelerated [39]. This recrystallized grain refinement will facilitate the Hall-Petch strengthening mechanism in nanocomposites. On the other hand, the critical shear stress of the magnesium alloy related to Orowan strengthening due to the precipitation of the second phase can be expressed as [40,41]:

(2) $$\Delta \tau_{Orowan}=\frac{Gb}{2\pi \lambda \sqrt{1-\nu }}\ln(\frac{d_{p}}{b})$$

where G is the shear modulus, b is the Burgers vector, ν is the Poisson’s ratio, λ is the effective plane spanning the obstacle spacing, $\lambda =((0.953/\sqrt{f_{v}})-1)d_{p}$. $f_{v}$ and $d_{p}$ stand for the average volume fraction and size of the second phase, respectively. The effect of the second phase precipitation on the Orowan strengthening of magnesium alloy can be expressed as [41]:

(3) $$\Delta \sigma_{Orowan}=M\Delta \tau_{Orowan}$$

where M is the Taylor factor. When the size of the second phase is large, the crack tends to nucleate around the second phase and stress concentration is easy to produce, resulting in premature fracture of the sample during the tensile test. According to Equation (3), when the precipitated phases Mg_17_Al_12_ is relatively coarse, the Orowan strengthening caused by the dispersion of the second phase would be reduced. On the other hand, it is prone to generate stress concentration and crack within the zone where the phase Mg_17_Al_12_ precipitates, which decrease the elongation of the composite during the tensile test at room temperature. In addition, when nano-sized precipitated phases are obtained in the composite the Orowan strengthening would be enhanced based on Equation (3). It is worth noting that due to the large cumulative strain of multi-pass forging in the current study, the dislocation density between the second phase Mg_17_Al_12_ and the matrix is high and the lattice mismatch exists both in the alloy and composite [42]. Thus, the precipitated second phase is mainly spherical. On the other hand, the increased lattice defects in the matrix during multi-pass forging could promote the non-uniform nucleation of the precipitated phase Mg_17_Al_12_, accelerate the diffusion of solid solution atoms in the matrix and effectively reduce the time for the nucleation and growth of the second phase, which is also beneficial to the precipitation of the spherical second phase. Therefore, it is very important to control the size and distribution of the precipitated second phase in order to achieve high strength magnesium matrix composites containing nano-sized particles.

#### 3.3.4. Effect of Nanoparticle Distribution on the Strength-Toughness Properties

The addition to reinforcement, nanoparticles can also affect the dynamic recrystallization behavior of the matrix alloy [43]. When the particle size is less than 1 μm, the particles would not promote the nucleation of dynamic recrystallization for the matrix during hot extrusion [44]. Therefore, the dispersed SiC nanoparticles in the current study cannot promote dynamic recrystallization nucleation, but produce a Zenner pinning effect on the matrix grains during the multi-pass forging [44,45], hindering the growth of the dynamic recrystallized grains. This is beneficial to improve the mechanical properties of the composite according to Hall-Petch strengthening.

The distribution of SiC nanoparticles also has an effect on the Orowan strengthening. When the distribution of SiC nanoparticles is relatively uniform, since the size and spacing of the nanoparticles is small, the dislocations need to bypass the nanoparticles using Orowan bowing. In the previous study, the grain size of nano-SiCp/AZ91 composites after multi-pass forging at a constant-temperature of 400 °C was increased significantly compared with that at 350 °C, but the yield strength increased, which can be attributed to that of the improved distribution of SiC nanoparticles for the composite deformed at higher temperature can promote the Orowan strengthening. As the passes and temperatures of the multi-pass forging varies, the yield strength of the composite after nine passes (400-6P + 350-3P) and 12 passes (400-6P + 350-3P + 300-3P) is higher than that after six passes (400-3P + 350-3P) and nine passes (400-3P + 350-3P + 300-3P), which is also associated with the Hall-Petch strengthening caused by the improved nanoparticle distribution on the recrystallized grain refinement and the increase in the Orowan strengthening. However, a small number of dense-nanoparticles zones still exist in the composites after multi-pass forging under different process conditions. The aggregation of these SiC nanoparticles in the dense-nanoparticles zones lead to the premature cracking of the composite during the tensile test at room temperature, decreasing the tensile strength and elongation.

## 4. Conclusions

In this study, both AZ91 alloy and nano-SiCp/AZ91 composite were processed by multi-pass forging under varying passes and temperatures. By comparing the microstructure and mechanical properties between the alloy and the composite, the following conclusions can be obtained:

(1) Complete recrystallization occurs in both the AZ91 alloy and composite after six passes of multi-pass forging at a constant temperature of 400 °C. Further refinement of dynamic recrystallized grains and dynamic precipitates exist as the temperature decreases and pass increases.
(2) Under the same multi-pass forging condition, the grain size of the nano-SiCp/AZ91 composite is smaller than that of the AZ91 alloy, which can be attributed to the pinning effect of SiC nanoparticles on the grain boundaries.
(3) The texture intensity for the 12 passes of multi-pass forging with varying temperatures is increased compared with that after 9 passes. In contrast, there is no significant difference in the average values for schmid factors between the nano-SiCp/AZ91 composites after multi-pass forging for nine passes and for 12 passes.
(4) Due to the Orowan strengthening effect and grain refinement strengthening resulting from externally applied SiC nanoparticles and internally precipitated second phases, the yield strength of the composite is higher than that of the AZ91 alloy under the same conditon of multi-pass forging.
(5) The strength-toughness properties of the nano-SiCp/AZ91 composites are related to matrix grain size, texture evolution, SiC nanoparticles distribution and the precipitated second phases based on the comparison between the AZ91 alloy and the composite.

## Figures and Tables

**Table 1:** The mechanical properties of AZ91 alloy and nano-SiCp/AZ91 composites.

Materials	Yield Strength (MPa)	Ultimate Tensile Strength (MPa)	Elongation (%)
AZ91-6P	158 ± 4.1	292 ± 7.6	7.8 ± 0.7
AZ91-9P	178 ± 5.4	257 ± 6.4	2.6 ± 0.3
AZ91-12P	175 ± 5.2	286 ± 7.1	11 ± 0.9
nano-SiCp/AZ91-6P	194 ± 5.7	300 ± 7.8	9.2 ± 0.6
nano-SiCp/AZ91-9P	217 ± 6.2	289 ± 7.3	2.5 ± 0.2
nano-SiCp/AZ91-12P	206 ± 5.9	285 ± 6.9	3.3 ± 0.4
